# Smartphone application-based rehabilitation in patients with chronic respiratory and cardiovascular diseases

**DOI:** 10.1038/s41598-024-53583-2

**Published:** 2024-02-06

**Authors:** Chiwook Chung, Ah-Ram Kim, Dongbum Kim, Hee Kwon, Seong Ho Lee, Il-Young Jang, Min-Woo Jo, Do-Yoon Kang, Sei Won Lee

**Affiliations:** 1 Department of Pulmonary and Critical Care Medicine, Asan Medical Center, University of Ulsan College of Medicine, 88 Olympic-ro 43-gil, Songpa-gu, Seoul, 05505 Republic of Korea; 2 Department of Pulmonary and Critical Care Medicine, Gangneung Asan Hospital, University of Ulsan College of Medicine, Gangneung, Republic of Korea; 3 Department of Cardiology, Asan Medical Center, University of Ulsan College of Medicine, 88 Olympic-ro 43-gil, Songpa-gu, Seoul, 05505 Republic of Korea; 4 LifeSemantics Corp., Seoul, Republic of Korea; 5 Division of Geriatrics, Department of Internal Medicine, Asan Medical Center, University of Ulsan College of Medicine, Seoul, Republic of Korea; 6 Department of Preventive Medicine, Asan Medical Center, University of Ulsan College of Medicine, Seoul, Republic of Korea

**Keywords:** Cardiology, Health care

## Abstract

Rehabilitation improves symptoms, quality of life, and survival in patients with chronic respiratory or cardiovascular disease. We evaluated smartphone application-based rehabilitation programs for patients with chronic respiratory or cardiovascular diseases. This was a single-center prospective single arm study. Participants underwent smartphone application-based pulmonary or cardiac rehabilitation for 12 weeks. A total of 93 participants were recruited, and 75 visited after rehabilitation. Their median age was 67.0 (interquartile range, 60.0–70.8) years, and 60 (80.0%) were men. For patients with chronic respiratory disease (n = 41), VO_2_peak (median 13.7 to 15.4 ml/kg/min, *P* = 0.049), chronic obstructive pulmonary disease assessment test (median 14 to 6, *P* < 0.001), Euro-QoL 5-Dimension 5-Level (EQ-5D-5L) index (median 0.795 to 0.862, *P* = 0.001), and Health-related Quality of Life Instrument with 8 Items (HINT-8) index (median 0.784 to 0.855, *P* < 0.001) were significantly improved. For patients with chronic cardiovascular disease (n = 34), VO_2_peak (median 21.8 to 23.3, *P* = 0.007), EQ-5D-5L index (median 0.871 to 1.000, *P* = 0.037), and HINT-8 index (median 0.890 to 0.903, *P* < 0.001) were significantly improved. The smartphone application-based rehabilitation program improved exercise capacity and quality of life in patients with chronic respiratory or cardiovascular disease.

**Trial registration**: https://clinicaltrials.gov/ct2/show/NCT05383950 (20/05/2022).

## Introduction

Globally, chronic respiratory and cardiovascular diseases remain important causes of mortality and morbidity^1–3^. In 2019, lung cancer, chronic obstructive lung disease (COPD), lower respiratory infections and ischemic heart disease are among the top-ten causes of disability-adjusted life-years among people over 50 years of age^1^. Individuals with chronic respiratory and cardiovascular diseases also experience various problems, including reduced exercise capacity and poor quality of life^4–8^.

Pulmonary rehabilitation is an intervention that can improve physical and psychological conditions of individuals with chronic respiratory disease via exercise training, behavior modification, and education^5^. Pulmonary rehabilitation has been shown to efficaciously improve the exercise capacity, dyspnea, and quality of life in patients with chronic respiratory disease^4,5,9^. Furthermore, muscle wasting and muscle dysfunction are common in chronic respiratory diseases, and result in decreased respiratory function and exercise capacity^10,11^. For the population affected by these conditions, pulmonary rehabilitation with exercise training and nutritional support can be the most effective alternative intervention^11^.

Cardiac rehabilitation is a component of a comprehensive treatment program that includes exercise training, medical treatment, and education to improve mortality and quality of life as well as reduce recurrence of individuals with chronic cardiovascular disease^6–8^. Increased exercise capacity has been found to be correlated with reduced mortality risk^12^, and increased physical activity was associated with reduced mortality risk in individuals with cardiovascular disease^13^.

However, participating in center-based rehabilitation has remained challenging for patients with chronic respiratory and cardiovascular diseases due to transportation barriers, lack of facilities and motivation, and low social support^14–16^. Recently, evidence supporting the effectiveness and feasibility of home-based pulmonary and cardiac rehabilitation compared with center-based rehabilitation has increased^17,18^. Furthermore, obstacles to practicing face-to-face rehabilitation programs during the COVID-19 pandemic have prompted the development of telerehabilitation alternatives to center-based rehabilitation for chronic respiratory and cardiovascular disease treatment^19,20^. Therefore, we developed a smartphone application and aimed to evaluate the efficacy of smartphone application-based rehabilitation programs to improve exercise capacity and quality of life of patients with chronic respiratory or cardiovascular disease.

## Methods

### Study design

This was a single-center prospective single arm study designed to evaluate the clinical efficacy of smartphone application-based rehabilitation in patients with chronic respiratory or cardiovascular disease. In 2022, 90 participants from Asan Medical Center, comprising 50 and 40 patients with chronic respiratory and cardiovascular disease, respectively, were recruited. Participants were screened at outpatient clinics of the pulmonology and cardiology departments. Subsequently, they were assigned to the pulmonary and cardiac rehabilitation program, respectively.

Participants were provided with the smartphone application and performed an application-based self-directed rehabilitation program for the entire intervention duration of 12 weeks. They were evaluated at the baseline and at the end of the rehabilitation.

The study protocol was approved by the Institutional Review Board of Asan Medical Center (Approval number: 2022-0562). Written informed consent was obtained from all participants prior to inclusion. This study complied with the guidelines stipulated in the Declaration of Helsinki and all methods were performed in accordance with the relevant guidelines. Finally, this study was registered in the ClinicalTrials.gov database (NCT05383950, https://clinicaltrials.gov/ct2/show/NCT05383950, 20/05/2022).

### Study participants

Patients with a clinically diagnosed chronic respiratory or cardiovascular disease were recruited at the outpatient clinic of Asan Medical Center. The inclusion criteria were as follows: (1) aged 20–80 years; (2) dyspnea score ≥ 1 in the modified Medical Research Council (mMRC) or scale ≥ I in the New York Heart Association Functional Classification (NYHA); and (3) had a chronic respiratory or cardiovascular disease and underwent regular medications. Chronic respiratory diseases included (1) obstructive lung disease, such as asthma and COPD (defined as exhibiting a forced expiratory volume in one second [FEV_1_] < 80% of the predicted value or a FEV1/forced vital capacity [FVC] < 0.7), (2) bronchiectasis (defined as bronchiectasis visualized in more than one lobe of the lungs via chest computed tomography), or (3) restrictive lung disease, such as tuberculous lung destruction and interstitial lung disease (defined as an FVC or diffusing capacity for carbon monoxide [DL_CO_] < 80% of the predicted value)^21^. Chronic cardiovascular diseases included (1) ischemic heart disease (defined as coronary artery reperfusion therapy for myocardial infarction or angina pectoris) and (2) heart failure (defined as a left ventricular ejection fraction < 50% as measured by echocardiography)^22^. The exclusion criteria were as follows: (1) an acute exacerbation of underlying disease within 4 weeks immediately before enrollment; (2) inability to perform the rehabilitation program due to disability; and (3) inability to run the smartphone application.

### Smartphone application and rehabilitation program

**Figure 1:**
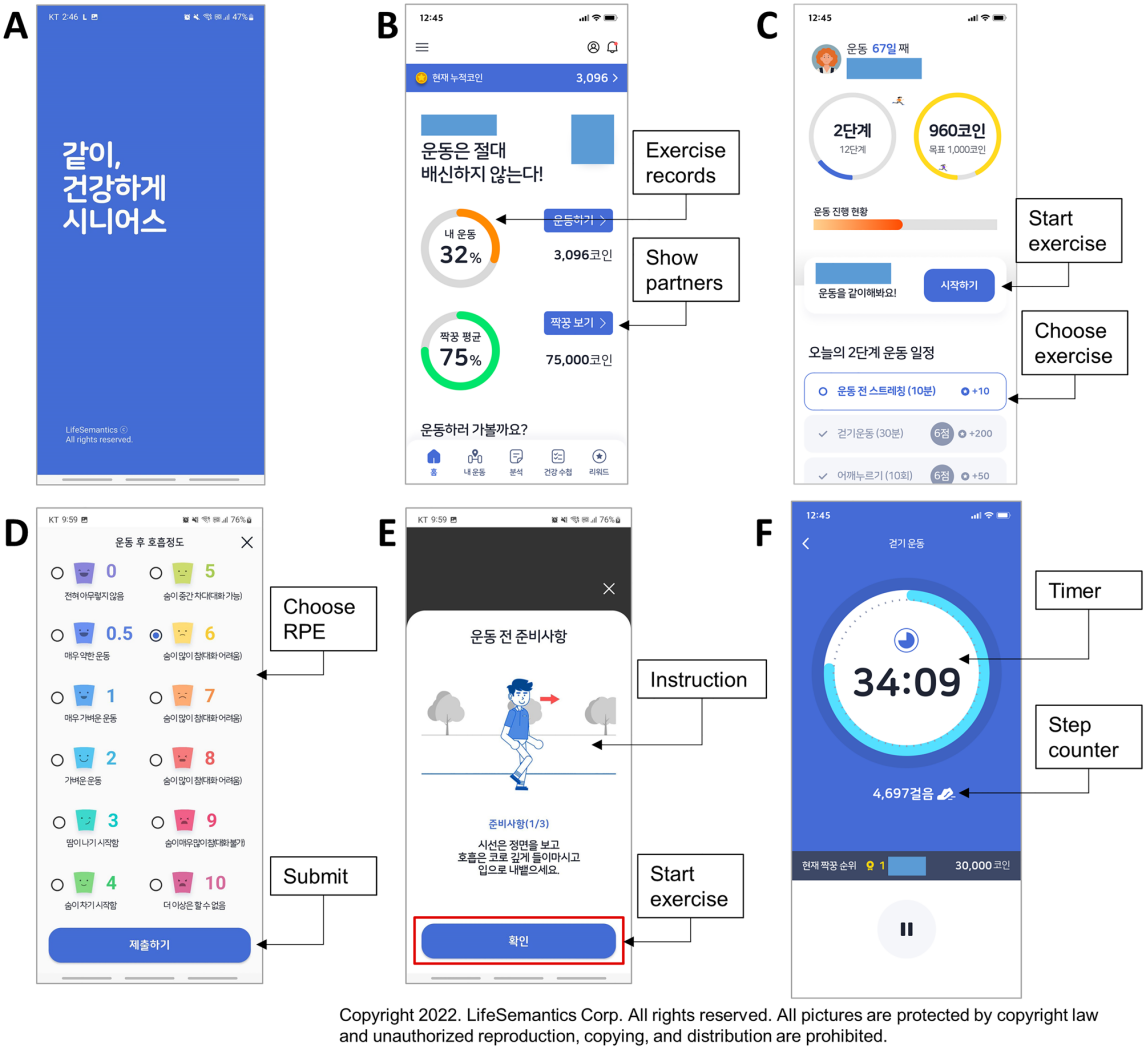
Screenshots of the “SENIORS” application. Shown are: (**A**) Opening screen (**B**) Home menu, which displays daily and total exercise records of the user and an exercise partner. (**C**) Each exercise program could be selected in a daily exercise schedule menu. (**D**) Rating of perceived exertion (RPE) scale, which were evaluated after each exercise. (**E**) Instructions for walking exercises. (**F**) Step counter and timer for walking exercises.

The smartphone application (SENIORS) was developed by LifeSemantics Corp. (Seoul, Republic of Korea). Briefly, investigators reviewed existing smartphone applications, the relevant scientific literature, and rehabilitation guidelines to design an application and related rehabilitation programs^4–6,23,24^. The application was developed on the Android platform (requiring at least Android 8.0). The application provides exercise programs, records, and partners, and disease education (Fig. 1). Pulmonary rehabilitation comprises one level of the exercise program and cardiac rehabilitation comprises three levels of the exercise program and includes different anaerobic exercises. Each exercise program consisted of two 30-min periods of aerobic and anaerobic exercise, respectively. Participants practiced various core and limb muscle exercises, and the exercise level increased weekly (Supplementary Table 1). Finally, participants could earn rewards that depended on their exercise records.

### Study outcome

The primary outcome was peak oxygen uptake (VO_2_peak) as measured by the cardiopulmonary exercise test after the end of rehabilitation^25^. A cardiopulmonary exercise test was performed based on incremental protocol^25^. For patients with chronic respiratory disease, cycle ergometer (VIAsprint 150P; Carefusion, San Diego, CA, USA) and metabolic cart (Vmax 29; SensorMedics, Yorba Linda, CA, USA) were used. For patients with chronic cardiovascular disease, treadmill (COSMED T150 DE; h/p/cosmos sports & medical, Nussdorf-Traunstein, Germany) and metabolic cart (Quark CPET; COSMED, Rome, Italy) were used. Secondary outcomes after the end of rehabilitation included dyspnea scores, responses to quality of life questionnaires, lung function (for chronic respiratory disease), exercise time, and metabolic equivalents (METs) during a cardiopulmonary exercise test (for chronic cardiovascular disease), and a limb muscle test. Dyspnea symptoms were assessed using the mMRC dyspnea scale (for chronic respiratory disease) and NYHA class (for chronic cardiovascular disease). Quality of life questionnaires included the Euro-QoL 5-Dimension 5-Level (EQ-5D-5L)^26,27^, Health-related Quality of Life Instrument with 8 Items (HINT-8) questionnaire^28–30^, and COPD assessment test (CAT, for chronic respiratory disease)^31^. EQ-5D-5L and HINT-8 index scores were calculated based on previous studies^29,30,32^. Lung function was quantified by FVC, FEV1, and DL_CO_^21^. The limb muscle tests included hand grip strength and limb muscle mass as measured by bioelectrical impedance analysis^33,34^.

### Sample size calculation

The sample size was calculated to determine the significance of improvements in the primary outcome between baseline and after rehabilitation based on previous studies. For pulmonary rehabilitation, a previous study demonstrated that baseline and after rehabilitation VO_2_peak measurements had mean values of 13.2 ± 3.0 and 14.8 ± 4.1 ml/kg/min, respectively^35^. We assumed that the participants’ mean baseline VO_2_peak was 13.2 ± 3.0 ml/kg/min, and therefore resulted in a 10% increase after rehabilitation. To achieve an alpha of 0.05 and a power of 80%, at least 41 participants were required. Moreover, to allow a 20% drop-out, 50 participants were required. For cardiac rehabilitation, we noted that a previous study demonstrated that the mean baseline and after rehabilitation VO_2_peak values were 22.8 ± 4.2 and 26.0 ± 4.0 ml/kg/min, respectively^36^. Therefore, we assumed that the participant mean baseline VO_2_peak was 23.0 ± 4.0 ml/kg/min, resulting in a 10% increase after rehabilitation. To achieve an alpha of 0.05 and a power of 80%, at least 31 participants were required. To account for an anticipated drop-out rate of 20%, 40 participants were required.

### Statistical analysis

Continuous variables were presented as medians [interquartile range (IQR)] and were compared using Wilcoxon signed rank tests. Categorical variables were presented as counts (percentages). All *P*-values were two-tailed, with the threshold of statistical significance set to *P* < 0.05. All statistical analyses were performed using SPSS version 26.0 (Statistical Package for the Social Sciences, IBM SPSS Corporation, Armonk, NY, USA).

## Results

### Participant baseline characteristics

**Figure 2:**
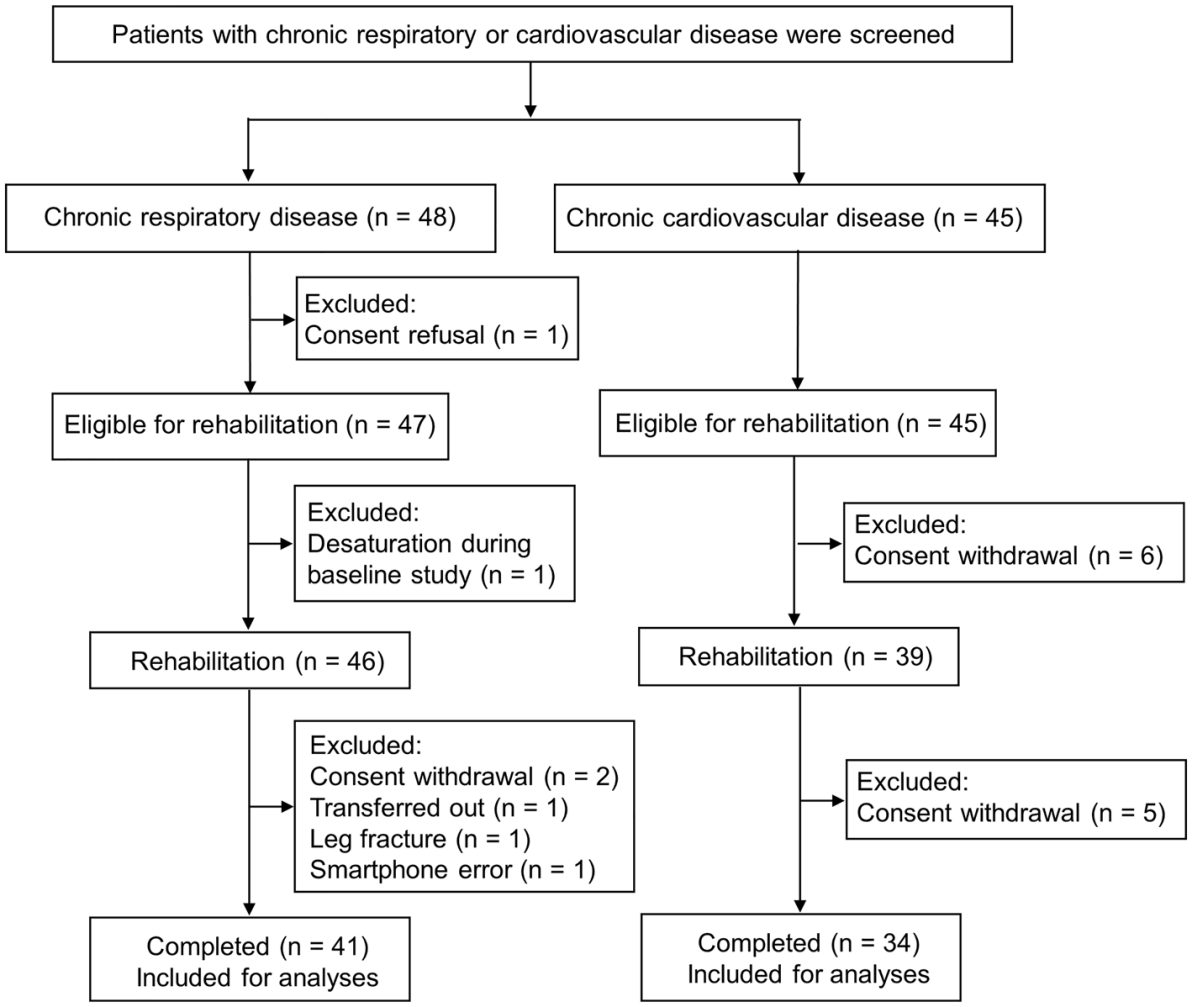
Study flowchart. One patient in the chronic respiratory disease group experienced a leg fracture; however, this was unrelated to the rehabilitation.

Figure 2 shows the flowchart of the study process. A total of 93 participants were recruited (48 and 45 from the pulmonary and cardiac departments, respectively), and 85 started rehabilitation (46 and 39 from the pulmonary and cardiac departments, respectively). Finally, 75 (41 and 34 from the pulmonary and cardiac departments, respectively) visited after rehabilitation, resulting in 20% withdrawal rate^37,38^. Table 1 shows the baseline characteristics of the study participants. Their median age was 67.0 (IQR, 60.0–70.8) years and 60 (80.0%) were men. Fifty-six (74.7%) participants had a history of smoking. Of the 41 participants with chronic respiratory disease, 33 had obstructive lung disease and eight had bronchiectasis. All 34 participants with chronic cardiovascular disease had ischemic heart disease.

**Table 1:** Baseline characteristics of participants.

	Total (n = 75)	Chronic respiratory disease (n = 41)	Chronic cardiovascular disease (n = 34)
Age (years)	67.0 [60.0–70.8]	67.0 [62.0–73.0]	64.5 [55.5–69.3]
Male	60 (80.0)	32 (78.0)	28 (82.4)
Body weight (kg)	68.0 [59.0–73.8]	64.8 [54.3–72.6]	70.0 [64.1–74.6]
Height (cm)	168.0 [162.0–171.8]	167.0 [159.5–171.0]	169.0 [165.0–172.0]
BMI (kg/m^2^)	24.1 [21.6–25.8]	23.7 [20.2–26.2]	24.3 [22.5–25.6]
Ever-smoker	56 (74.7)	29 (70.7)	27 (79.4)
Underlying disease			
Diabetes mellitus	14 (18.7)	3 (7.3)	11 (32.4)
Hypertension	31 (41.3)	15 (36.6)	16 (47.1)
Dyslipidemia	28 (37.3)	12 (29.3)	16 (47.1)
Malignancy	6 (8.0)	4 (9.8)	2 (5.9)

Data are presented as median [interquartile range] or count (%), unless otherwise indicated.

BMI, body mass index.

### Clinical parameters of participants

**Figure 3:**
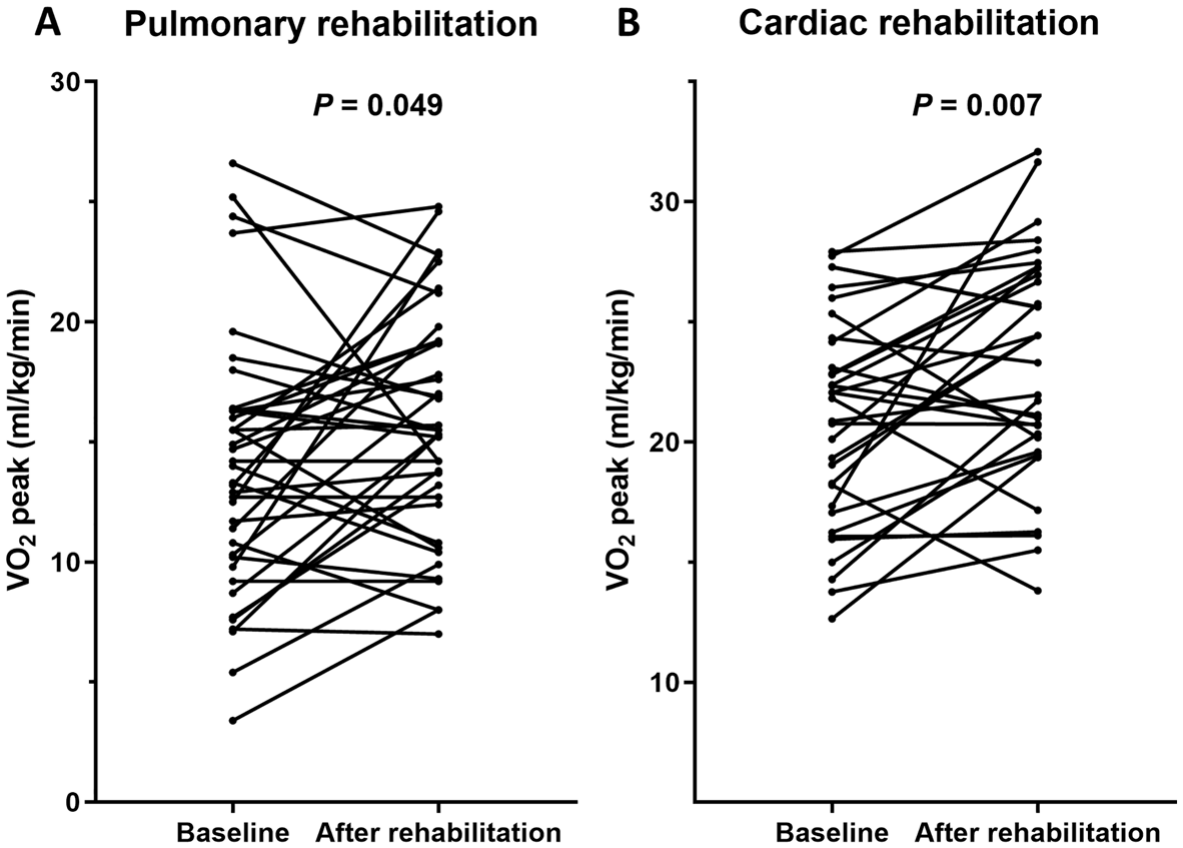
Change in peak oxygen uptake (VO_2_peak) of participants. Data are shown for: (**A**) Pulmonary rehabilitation and (**B**) cardiac rehabilitation.

Among participants with chronic respiratory diseases, their VO_2_peak was significantly improved (*P* = 0.049). Moreover, we observed significant improvement in CAT score (*P* < 0.001), EQ-5D-5L index (*P* = 0.001), and HINT-8 index (*P* < 0.001; Table 2, Fig. 3). Among participants with chronic cardiovascular disease, we observed significant improvement in VO_2_peak (*P* = 0.007) and METs (*P* = 0.011). Significant improvement was also evident in the duration (exercise time) of the cardiopulmonary exercise test (*P* < 0.001), EQ-5D-5L index (*P* = 0.037), and HINT-8 index (*P* < 0.001; Table 3, Fig. 3). No participants experienced disease exacerbation or musculoskeletal injury related to rehabilitation activities during the study period.

**Table 2:** Clinical parameters of participants with chronic respiratory disease.

n = 41	Baseline	After rehabilitation	*P* value
VO_2_peak (ml/kg/min, n = 38)^a^	13.7 [10.1–16.3]	15.4 [12.0–19.2]	0.049
mMRC dyspnea scale	2 [1–2]	1 [1–2]	0.062
CAT score	14 [10–20]	6 [3–9]	< 0.001
EQ-5D-5L index	0.795 [0.724–0.862]	0.862 [0.808–1.000]	0.001
HINT-8 index	0.784 [0.711–0.825]	0.855 [0.803–0.895]	< 0.001
FEV1 (%predicted)	61.0 [32.0–72.0]	50.0 [33.0–71.5]	0.031
FVC (%predicted)	77.0 [64.5–90.0]	75.0 [64.5–85.0]	0.021
DL_CO_ (%predicted, n = 40)	56.5 [40.3–73.5]	55.5 [41.3–68.5]	0.265
Hand grip strength (kg)	39.0 [31.0–46.0]	40.0 [34.0–46.0]	0.442
Limb muscle mass (kg)	19.3 [17.0–22.2]	20.2 [17.5–22.3]	0.081
Upper limb	4.7 [3.8–5.7]	5.1 [4.1–5.9]	< 0.001
Lower limb	14.8 [12.7–16.5]	14.9 [12.7–16.6]	0.510

Data are presented as median [interquartile range].

VO_2_peak, peak oxygen uptake; mMRC, mMRC Modified Medical Research Council; CAT, COPD Assessment Test; EQ-5D-5L, Euro-QoL 5-Dimension 5-Level; HINT-8, Health-related Quality of Life Instrument with 8 Items; FEV1, forced expiratory volume in one second; FVC, forced vital capacity; DL_CO_, diffusing capacity for carbon monoxide.

^a^VO_2_peak was measured using a cardiopulmonary exercise test.

**Table 3:** Clinical parameters of participants with chronic cardiovascular disease.

n = 34	Baseline	After rehabilitation	*P* value
VO_2_ peak (ml/kg/min, n = 33)^a^	21.8 [17.2–24.2]	23.3 [19.9–27.1]	0.007
Exercise time (sec, n = 33)^a^	513 [328–550]	555 [508–586]	< 0.001
METs (n = 33)^a^	6.2 [5.1–7.3]	6.5 [5.8–7.9]	0.011
NYHA class	1 [1–1]	1 [1–1]	> 0.999
EQ-5D-5L index	0.871 [0.802–1.000]	1.000 [0.840–1.000]	0.037
HINT-8 index	0.890 [0.812–0.908]	0.903 [0.859–1.000]	< 0.001
Hand grip strength (kg)	32.5 [27.6–40.0]	34.0 [24.8–42.0]	0.386
Limb muscle mass (kg, n = 32)	22.9 [20.3–24.8]	23.2 [21.0–24.8]	0.258
Upper limb	6.1 [5.2–6.6]	6.2 [5.5–6.9]	0.044
Lower limb	16.6 [15.2–17.8]	16.8 [15.3–18.2]	0.575

Data are presented as median [interquartile range].

VO_2_ peak, peak oxygen uptake; METs, Metabolic equivalents of task mMRC; NYHA, New York Heart Association; EQ-5D-5L, Euro-QoL 5-Dimension 5-Level; HINT-8, Health-related Quality of Life Instrument with 8 Items.

^a^VO_2_ peak, exercise time, and METs were measured using a cardiopulmonary exercise test.

### Participant compliance during rehabilitation

**Figure 4:**
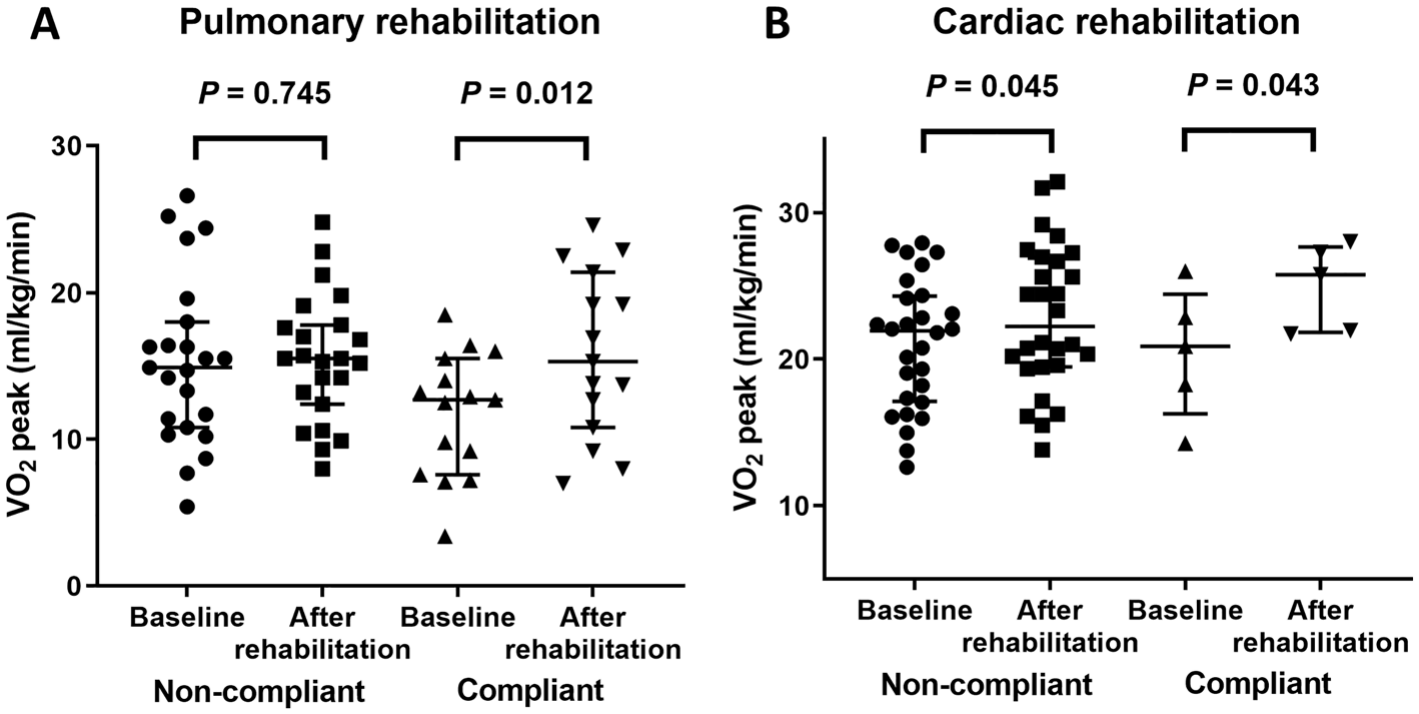
Change in peak oxygen uptake (VO_2_peak) of participants according to compliance. Data are shown for: (**A**) Pulmonary rehabilitation and (**B**) cardiac rehabilitation.

Participants who performed both aerobic and anaerobic exercises ≥ 30% of the entire study period of 84 days, based on the log data of application, were considered to be compliant participants. Among participants with chronic respiratory disease, 17 (41.5%) were compliant (aerobic exercise, median 19.0 days [IQR, 1.0–47.3] and anaerobic exercise, median 36.0 days [IQR, 2.8–56.3]). We observed significant improvement in VO_2_peak only in these compliant participants (*P* = 0.012). Among participants with chronic cardiovascular disease, only 5 (14.7%) were compliant (aerobic exercise, median 4.0 days [IQR, 0.0–21.0] and anaerobic exercise, median 3.5 days [IQR, 1.0–21.0]), and significant improvement in VO_2_peak was observed regardless of compliance (Fig. 4).

### HINT-8 distribution

To evaluate which dimensions of quality of life improved during rehabilitation, we subsequently analyzed the distribution of HINT-8 results by item and level. Participants with chronic respiratory disease reported significant improvements in all dimensions measured by HINT-8 except for climbing stairs. Participants with chronic cardiovascular disease reported significant improvement in terms of vitality, depression, and memory (Supplementary Table 2).

### Ease-of-use of the application

Approximately 80% of participants indicated that they perceived the application as easy to use (i.e., “very easy,” 68.0% of participants; “easy,” 14.7% of the participants) and were accustomed to using the application within 3 days (i.e., 64.0% and 21.3% within 1 and 3 days, respectively). Moreover, approximately two-thirds (65.3%) of the participants indicated that they wanted to use the application if it were commercially available. The most attractive point of the application was that it was a physician-designed exercise program (as indicated by 37.3% of respondents, Supplementary Table 3).

## Discussion

In this study, we evaluated a smartphone application-based rehabilitation program for patients with chronic respiratory and cardiovascular diseases. We found that the smartphone application-based rehabilitation program improved the clinical outcomes of participants, including exercise capacity and quality of life. Furthermore, older adult patients with chronic diseases can easily perform the rehabilitation program. Thus, smartphone application-based rehabilitation may be a useful treatment option for older adult patients with chronic diseases.

Exercise capacity and physical activity are important prognostic indicators for patients with chronic respiratory and cardiovascular disease. For example, exercise capacity has been found to be an important predictor of mortality in patients with COPD^39^. In addition, low levels of physical activity were found to be correlated with high risks for disease exacerbation and mortality in patients with COPD^40^. Similarly, higher exercise capacity was correlated with reduced mortality risk in patients with chronic cardiovascular diseases^12^, while high levels of physical activity resulted in reduced mortality risk in patients with cardiovascular disease^13^. This study demonstrated that exercise capacity can improve via an application-based rehabilitation program in older adult patients with chronic disease. Unfortunately, physical activity levels, such daily step counts, were not measured in this study. This would be simple to implement since it could be measured using a smartphone-mounted pedometer. Thus, further development of the application is required to obtain this data.

We found that the application-based rehabilitation program was associated with significantly improved quality of life for all groups of participants. In particular, patients with chronic respiratory disease reported significant improvements in CAT score, a predictor of the severity of airflow limitation and acute exacerbation in patients with COPD^41,42^. By improving exercise capacity and quality of life through rehabilitation programs, clinical outcomes such as disease exacerbation may be improved. We noted that no patient experienced acute disease exacerbation during the study period. Therefore, further studies are needed to evaluate the effect of rehabilitation on acute exacerbation or mortality using long-term follow-up assessments performed after the end of rehabilitation.

As previously described, compliance to rehabilitation program is an important issue in home-based rehabilitation^43^. A previous study reported an estimated − 0.22 (95% CI, − 0.74–0.31) decrease in the CAT score in every 7-day increase in application use for pulmonary rehabilitation^44^. However, another study reported that as time passed, the number of smartphone application users undergoing pulmonary rehabilitation decreased^45^. Therefore, to steadily use the application and perform rehabilitation program, initial professional assessment and goal setting are important^46^. Enabling self-monitoring and self-evaluation, such as feedback using wearable device and adjustable exercise program, is also important in real-world practice^46^. Moreover, patients’ preferences should be considered in designing eHealth platforms to enhance user engagement^47^.

In this study, participants showed low levels of compliance, which was only 14.7% among participants with chronic cardiovascular disease. However, in the subgroup analysis, significant improvement in VO_2_peak was noted in compliant participants with chronic pulmonary disease. In a previous study of home-based pulmonary rehabilitation without supervision, participants with good compliance showed significant improvement in clinical indices compared with non-compliant participants^48^. Because the lack of motivation was an important factor for poor compliance in home-based rehabilitation^43^, attending physicians should emphasize the need for patients to steadily use the application and perform rehabilitation program to achieve significant clinical improvement. The first step of pulmonary and cardiac rehabilitation involves fostering awareness among patients that engaging in appropriate exercise can contribute to the improvement of their symptom. In this regard, the application guided rehabilitation would be helpful. Moreover, some methods, such as regular text messages or telephone contacts from health care, can be applied to enhance motivation^49^. Repeated exposure to exercises that promote the use of the application may motivate individuals to exercise more.

In this study, the vitality dimension quantified by HINT-8 was reported as having improved significantly for all participant groups. The rehabilitation program required walking outdoors for 30 min daily, and this may have encouraged the participants to engage in outdoor activities. Previous studies have demonstrated that exercise is associated with improved vitality in patients with chronic diseases^50,51^. Older adult patients with chronic diseases may be sedentary and prefer to remain indoors, and therefore a significant improvement in vitality may be realized via daily walks outdoors. Taken together, our data suggest that increased physical and outdoor activity may be an important factor improving participant quality of life in this study.

Interestingly, the memory and depression dimensions of HINT-8 improved in all participant groups. There is considerable scientific evidence that exercise can improve the performance of memory systems, even in older adults^52,53^. For example, one study showed that aerobic exercise increased the volume of gray and white matter in the prefrontal cortices of older adults^54^. Exercise has also been shown to increase blood volume, perfusion, and volume of the hippocampus in older adults^55,56^. In addition to structural changes in the brain, previous studies have also demonstrated that exercise can improve cognitive performance and functional connectivity in the brain^57,58^. Thus, physical activity is thought to improve cognitive function, improve memory, induce antidepressant effects, and confer a sense of wellbeing^59^. Further studies are required to ascertain the associations between physical exercise and mental health in patients with chronic respiratory and cardiovascular disease.

This study has notable strengths. Despite most participants being older adult, the application-guided rehabilitation treatment showed that they can achieve a significant improvement in exercise capacity and quality of life, particularly with respect to their mental health. Over 80% of participants perceived that the application was easy to use and became familiar with it within a remarkably short period of time. Furthermore, we did not observe disease exacerbation or musculoskeletal accidents during the study period. Previous studies also reported that adverse event rates were acceptable during home-based pulmonary and cardiac rehabilitation^60,61^. These results therefore highlight the fact that smartphone application-based rehabilitation can be successfully performed even in older adult patients with chronic diseases.

This study has some implications for further research. Although we observed significant improvement in some clinical parameters of the participants, further studies with additional participants and a randomized controlled study design are required to ascertain the efficacy of smartphone application-based rehabilitation programs. Although we noted that no patient experienced acute disease exacerbation during the study period (12 weeks), further studies are needed to evaluate the effect of rehabilitation on acute exacerbation or mortality using long-term follow-up assessments performed after the end of rehabilitation.

This study has some limitations. Physical activity levels, such daily step counts, were not measured in this study owing to the limitation of application. Thus, further development of the application is required to obtain this data through a smartphone-mounted pedometer. Moreover, this study failed to demonstrate improvement in hand grip strength and limb muscle mass. Although the rehabilitation program provided anaerobic exercise with incremental intensity, nutritional support—such as protein supplementation—was not provided. Nutritional support to maintain adequate body mass index and muscle mass is an important component of rehabilitation in chronic disease^4,6^. Further studies with proper nutritional support are expected to improve muscle mass and strength in patients with chronic diseases.

In conclusion, the smartphone application-based rehabilitation program described here improved clinical outcomes, including exercise capacity and quality of life, in patients with chronic respiratory or cardiovascular diseases. Furthermore, older adult patients with chronic diseases could easily and safely perform smartphone application-based rehabilitation. Thus, smartphone application-based rehabilitation programs may be a useful treatment option for older adult patients with chronic diseases when center-based rehabilitation is not feasible.

### Supplementary Information

Supplementary Tables.

## Data Availability

The datasets used and analyzed during the current study are available from the corresponding author upon reasonable request.
