# Supplementary material for: Smartphone application-based rehabilitation in patients with chronic respiratory and cardiovascular diseases
Source: Sci Rep. 2024 Feb 6;14:3018. doi: 10.1038/s41598-024-53583-2 (PMC10847123; doi:10.1038/s41598-024-53583-2)
Supplement: Supplementary file 1 — Supplementary Tables. [file 41598_2024_53583_MOESM1_ESM.docx]

**Supplementary Table 1.** Content of anaerobic exercise program

| Warm-up stretching | Active ROM exercise | Latex resistance band exercise |
| --- | --- | --- |
| Scapulothoracic joint  Shoulder joint  Elbow joint  Wrist joint  Hip joint flexion  Hip joint extension  Hip joint external rotation  Knee joint  Ankle joint | Hand clap  Wall push-up  Wall lateral pull-down  Knee assisted push-up  Push-up  Ankle dorsiflexion  Ankle plantarflexion  Knee extension  Hip adduction  Glute bridge  Clamshell  Wall squat  Squat  Lunge | Band press  Upper back exercise  Lower back exercise  Monster walk |
| Dumbbell exercise | Cool-down stretching | Aerobic exercise |
| Biceps exercise  Triceps exercise  Pectoralis exercise  Deltoid exercise | Wrist and elbow  Shoulder  Neck  Hamstring and calf  Quadriceps | Walking exercise |

ROM: range of motion

**Supplementary Table 2.** HINT-8 distribution by item and level in participants.

| HINT-8 item | Chronic respiratory disease  (n = 41) | | | Chronic cardiovascular disease  (n = 34) | | |
| --- | --- | --- | --- | --- | --- | --- |
|  | Baseline | After rehabilitation | *P* value | Baseline | After rehabilitation | *P* value |
| Climbing stair | 2 [2-3] | 2 [2-3] | 0.072 | 1 [1-2] | 1 [1-1] | 0.405 |
| Pain | 1 [1-2] | 1 [1-1] | 0.005 | 1 [1-1] | 1 [1-1] | 0.083 |
| Vitality | 2 [2-3] | 1 [1-2] | <0.001 | 2 [1-3] | 1 [1-2] | 0.003 |
| Working | 2 [1-2] | 1 [1-2] | 0.001 | 1 [1-1] | 1 [1-1] | 0.180 |
| Depression | 1 [1-2] | 1 [1-1] | 0.022 | 1 [1-2] | 1 [1-1] | 0.020 |
| Memory | 2 [1-2] | 1 [1-2] | 0.002 | 2 [1-2] | 1 [1-2] | 0.013 |
| Sleep | 1 [1-2] | 1 [1-1] | 0.008 | 1 [1-2] | 1 [1-2] | 0.285 |
| Happiness | 2 [2-3] | 1 [1-3] | 0.003 | 2 [1-3] | 2 [1-3] | 0.644 |

Data are presented as median [interquartile range].

HINT-8, Health-related Quality of Life Instrument with 8 Items.

**Supplementary Table 3.** Application service evaluation questionnaire

| Question | N = 75 |
| --- | --- |
| How easy and convenient was the application service provided to you? |  |
| Very easy | 51 (68.0%) |
| Easy | 11 (14.7%) |
| Difficult | 11 (14.7%) |
| Very difficult | 2 (2.7%) |
| How long did it take to adapt to using the app after installation? |  |
| Within 1 day | 48 (64.0%) |
| Within 3 days | 16 (21.3%) |
| Within 1 week | 5 (6.7%) |
| Within 2 weeks | 1 (1.3%) |
| Difficult to use | 5 (6.7%) |
| Do you want to use the application service if it is commercialized? |  |
| Yes | 49 (65.3%) |
| No | 26 (34.7%) |
| If yes, why do you want to use it? (Multiple choice) |  |
| I think the app would be easy and fun to use. | 7 (13.5%) |
| If I have the app, I think I'll exercise more often. | 28 (53.8%) |
| I think exercising with others would be more effective than doing it alone. | 15 (28.8%) |
| I can get a reward if I exercise. | 2 (3.8%) |
| If no, why do you want not to use it (Multiple choice) |  |
| I think that the app is difficult and complex to use. | 2 (7.7%) |
| I don't need it because I'm still receiving rehabilitation and hospital treatment. | 4 (15.4%) |
| I prefer to exercise alone and not with others. | 12 (46.2%) |
| I can’t use my phone app very well. | 8 (30.8%) |
| What was the most attractive point of the application? |  |
| It is easier to use and understand than other apps. | 4 (5.3%) |
| It's fun to choose a partner to exercise with. | 2 (2.7%) |
| It's good to be able to check how much I've exercised. | 13 (17.3%) |
| Being able to get a reward helps me motivate myself. | 28 (37.3%) |
| It is good that the physicians designed the exercise and adjusted it for my condition. | 28 (37.3%) |

Data are presented as count (%).
